# Nrf2 deficiency does not affect denervation‐induced alterations in mitochondrial fission and fusion proteins in skeletal muscle

**DOI:** 10.14814/phy2.13064

**Published:** 2016-12-30

**Authors:** Yu Kitaoka, Kohei Takeda, Yuki Tamura, Shin Fujimaki, Tohru Takemasa, Hideo Hatta

**Affiliations:** ^1^ Department of Sports SciencesGraduate School of Arts and SciencesThe University of TokyoTokyoJapan; ^2^ Graduate School of Comprehensive Human ScienceUniversity of TsukubaTsukubaJapan

**Keywords:** Denervation, mitochondria, oxidative stress, skeletal muscle

## Abstract

Oxidative stress‐induced mitochondrial dysfunction is associated with age‐related and disuse‐induced skeletal muscle atrophy. However, the role of nuclear factor erythroid 2‐related factor 2 (Nrf2) during muscle fiber atrophy remains to be elucidated. In this study, we examined whether deficiency of Nrf2, a master regulator of antioxidant transcription, promotes denervation‐induced mitochondrial fragmentation and muscle atrophy. We found that the expression of Nrf2 and its target antioxidant genes was upregulated at 2 weeks after denervation in wild‐type (WT) mice. The response of these antioxidant genes was attenuated in Nrf2 knockout (KO) mice. Nrf2 KO mice exhibited elevated levels of 4‐hydroxynonenal in the skeletal muscle, whereas the protein levels of the mitochondrial oxidative phosphorylation complex IV was declined in the denervated muscle of these mice. Increased in mitochondrial fission regulatory proteins and decreased fusion proteins in response to denervation were observed in both WT and KO mice; however, no difference was observed between the two groups. These findings suggest that Nrf2 deficiency aggravates denervation‐induced oxidative stress, but does not affect the alterations in mitochondrial morphology proteins and the loss of skeletal muscle mass.

## Introduction

Skeletal muscle is a highly plastic tissue, and its disuse results in a decline of muscle mass and strength, accompanied by a decrease in mitochondrial content. Denervation is known as an effective animal model of muscle disuse; impaired muscle contractile function by the loss of innervation induces rapid loss of muscle mass and mitochondrial function (Wicks and Hood 1991). Previous studies reported that denervation enhanced mitochondrial reactive oxygen species (ROS) production and lipid peroxidation (O'Leary and Hood 2008; Abruzzo et al. 2010). This increased oxidative stress may play an important role in the adaptation of skeletal muscle to disuse, since some antioxidants have been reported to protect against immobilization‐induced muscle atrophy (Min et al. 2011; Talbert et al. 2013).

Nuclear factor erythroid 2‐related factor 2 (Nrf2) has been identified as the key regulator of antioxidant genes (Motohashi and Yamamoto 2004). Nrf2 binds to the antioxidant response element, leading to the transcriptional activation of its target antioxidant genes, such as catalase (Cat), heme oxygenase 1 (Hmox1), glutathione peroxidase 1 (Gpx1) (Muthusamy et al. 2012; Kitaoka et al. 2013). These antioxidants scavenge ROS and maintain intracellular redox homeostasis (Lee et al. 2005). Nrf2 protein content was found to be decreased in skeletal muscle of sedentary aged subjects with aging‐associated accretion of oxidative damage (Safdar et al. 2010). Similarly, Nrf2 signaling was impaired in the myocardium of aged mice (Gounder et al. 2012). Furthermore, a recent study showed that disruption of Nrf2 signaling aggravates cardiotoxin‐induced muscle damage (Shelar et al. 2016). In light of these findings, we hypothesized that Nrf2 deficiency enhances oxidative stress in denervated muscle and aggravates denervation‐induced muscle wasting.

Mitochondria are dynamic organelles, continuously remodeling through the process of fusion and fission to maintain the quality and function (Westermann 2012; Yan et al. 2012). Loss of mitochondrial fusion proteins causes not only severe mitochondrial dysfunction but also muscle atrophy (Chen et al. 2010). It has been demonstrated that exposure to ROS induced mitochondrial fragmentation in C2C12 myocytes (Fan et al. 2010; Iqbal and Hood 2014). Damaged and dysfunctional mitochondria are degraded by mitochondrial selective autophagy (mitophagy) (Gottlieb and Carreira 2010). Interestingly, the expression of p62, which plays essential roles for autophagic clearance of ubiquitinated proteins, is regulated by Nrf2 (Jain et al. 2010). These observations led us to hypothesize that Nrf2 deficiency negatively impacts mitochondrial quality control, and subsequently muscle function.

## Materials and Methods

### Animals and experimental design

Nrf2 knockout (KO) mice were obtained from Jackson Laboratory (Bar Harbor, ME). Mice were genotyped by polymerase chain reaction (PCR) analysis of tail DNA with the following primers: Nrf2 forward: 5‐GCCTGAGAGCTGTAGGCCC‐3, Nrf2 wild‐type (WT) reverse: 5‐GGAATGGAAAATAGCTCCTGCC‐3, Nrf2 mutant reverse: 5‐GACAGTATCGGCCTCAGGAA‐3. Animals were housed in an air‐conditioned room on a 12:12‐h light–dark cycle with standard chow and water ad libitum. Male WT C57/BL6 and Nrf2 KO mice at 10 weeks of age (*n* = 6 each genotype) underwent unilateral sciatic nerve transection surgery, as we described previously (Tamura et al. 2015). Briefly, mice were anesthetized using isoflurane, and a small incision was made in the posterior aspect of the left hindlimb to expose the sciatic nerve at the level of the femoral trochanter. A length of at least 5.0 mm of sciatic nerve was excised using small operating scissors. The skin was closed with surgical glue. The right hindlimb served as the sham‐operated control. Following 14 days of denervation, all mice were killed by cervical dislocation, and gastrocnemius muscles were quickly removed, snap‐frozen, and stored at −80°C. All experiments were approved by the Animal Experimental Committee of the University of Tokyo.

### RNA isolation and real‐time quantitative PCR

Approximately, 25 mg of gastrocnemius muscle was homogenized on ice in Trizol reagent (Life Technologies, Gaithersburg, MD), and then separated into organic and aqueous phases with chloroform. Total RNA was isolated using RNeasy Mini kit (Qiagen, Germantown, MD) according to the manufacturer's instructions, from the aqueous phase following precipitation with ethanol. RNA concentration was measured by spectrophotometry (Nanodrop ND1000, Thermo Scientific, Waltham, MA). First‐strand cDNA synthesis from 1 *μ*g of total RNA was performed with random hexamer primers using a high‐capacity cDNA reverse transcription kit (Applied Biosystems, Foster City, CA). The expression of Nrf2, Hmox1, Cat, Gclc (glutamate‐cysteine ligase catalytic subunit), Pgc‐1*α* (peroxisome proliferator‐activated receptor gamma coactivator 1‐alpha), Tfam (transcription factor A, mitochondrial), Cox4 (cytochrome c oxidase subunit IV), Cs (citrate synthase), Nd1 (NADH dehydrogenase subunit 1), Fis1 (fission, mitochondrial 1), Drp1 (dynamin‐related protein 1), Mfn1 (mitofusin 1), Mfn2, Opa1 (optic atrophy 1), Sqstm (p62), Park2 (parkin), Atg7 (autophagy‐related 7), and Map1lc3b (LC3) were quantified using the Thermal Cycler Dice Real‐Time System and SYBR Premix Ex taq II (Takara Bio, Shiga, Japan). All samples were run in duplicate simultaneously with a negative control that contained no cDNA. Tbp (TATA box‐binding protein) was used as a control housekeeping gene, the expression of which did not alter between groups. Forward and reverse primers for the aforementioned genes are shown in Table 1.

**Table 1:** Real‐time polymerase chain reaction primer sequences

Gene	Forward primer (5′‐3′)	Reverse primer (5′‐3′)
*Nrf2*	TTCTTTCAGCAGCATCCTCTCCAC	ACAGCCTTCAATAGTCCCGTCCAG
*Hmox1*	CACGCATATACCCGCTACCT	CCAGAGTGTTCATTCGAGCA
*Cat*	ACATGGTCTGGGACTTCTGG	CAAGTTTTTGATGCCCTGGT
*Gclc*	CAGTCAAGGACCGGCACAAG	CAAGAACATCGCCTCCATTCAG
*Pgc‐1α*	TTCCACCAAGAGCAAGTAT	CGCTGTCCCATGAGGTATT
*Tfam*	GAAGGGAATGGGAAAGGTAGA	AACAGGACATGGAAAGCAGAT
*Cox4*	CTCCAACGAATGGAAGACAG	TGACAACCTTCTTAGGGAAC
*Cs*	GCATGAAGGGACTTGTGTA	TCTGGCACTCAGGGATACT
*Nd1*	GTGGCTCATCTACTCCACTGA	TCGAGCGATCCATAACAATAA
*Fis1*	GCCTGGTTCGAAGCAAATAC	CACGGCCAGGTAGAAGACAT
*Drp1*	CGGTTCCCTAAACTTCACGA	GCACCATTTCATTTGTCACG
*Mfn1*	TTGCCACAAGCTGTGTTCGG	TCTAGGGACCTGAAAGATGGGC
*Mfn2*	GGGGCCTACATCCAAGAGAG	GCAGAACTTTGTCCCAGAGC
*Opa1*	GATGACACGCTCTCCAGTGAAG	CTCGGGGCTAACAGTACAACC
*Sqstm*	TGTGGTGGGAACTCGCTATAA	CAGCGGCTATGAGAGAAGCTAT
*Park2*	GTCTGCAATTTGGTTTGGAGTA	GCATCATGGGATTGTCTCTTAA
*Atg7*	TTTCTGTCACGGTTCGATAATG	TGAATCCTTCTCGCTCGTACT
*Map1lc3b*	GCTTGCAGCTCAATGCTAAC	CCTGCGAGGCATAAACCATGTA
*Tbp*	CTGCCACACCAGCTTCTGA	TGCAGCAAATCGCTTGGG

### Western blotting

Approximately, 25 mg of gastrocnemius muscle was homogenized in RIPA buffer (25 mmol/L Tris‐HCl, pH 7.6, 150 mmol/L NaCl, 1% NP‐40, 1% sodium deoxycholate, and 0.1% sodium dodecyl sulfate [SDS]) supplemented with protease inhibitor mixture (Complete Mini, ETDA‐free, Roche Applied Science, Indianapolis, IN) and phosphatase inhibitor mixture (PhosSTOP, Roche Applied Science). The total protein content of samples was quantified using the BCA protein assay (Pierce, Rockford, IL). Equal amounts of protein (10–15 *μ*g, depending on the protein of interest) were loaded onto 10% sodium dodecyl sulfate‐polyacrylamide gel electrophoresis (SDS‐PAGE) gels and separated by electrophoresis. Proteins were transferred to polyvinylidene difluoride membranes, and western blotting was carried out using primary antibodies against the following proteins: 4‐HNE (4‐hydroxynonenal; ab48506), Total OXPHOS Rodent WB Antibody Cocktail (ab110413), Fis1 (ab96764), Drp1 (ab56788), Mfn2 (ab124773), Parkin (ab77924) from Abcam (Cambridge, MA); Phospho‐Drp1 (Ser616, #3455), ubiquitin (#3933), total ULK1 (#8054), phospho‐ULK1 (Ser555, #5869 and Ser757, #6888), Hmox1 (#70081) from Cell Signaling Technology (Danvers, MA); Opa1 (#612606) from BD Transduction Laboratories (Tokyo, Japan); p62/SQSTM1 (PM045), LC3 (M152‐3) from MBL (Nagoya, Japan); Cat (sc‐271803) from Santa Cruz Biotechnology (Santa Cruz, CA). Ponceau staining was used to verify consistent loading.

### Statistical analysis

Data were expressed as mean ± standard error of means (SEM). Two‐way analysis of variance (ANOVA) (denervation × Nrf2) was performed, followed by Bonferroni multiple comparison test when an interaction was observed (GraphPad Prism 6.0, La Jolla, CA). Statistical significance was defined as *P* < 0.05.

## Results

### Body and muscle weight

**Figure 1:**
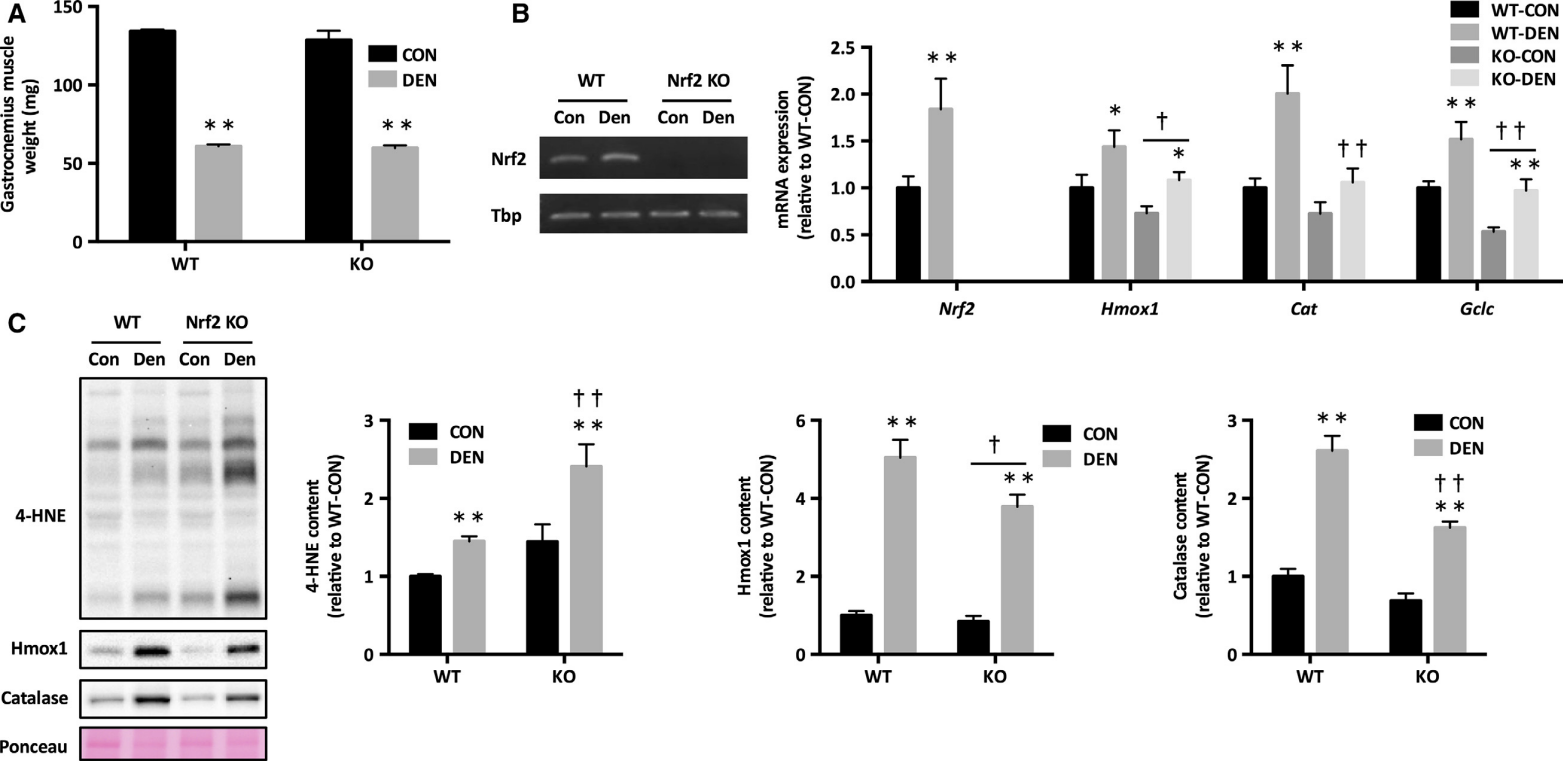
Oxidative stress and expression of antioxidant genes in response to denervation in Nrf2 KO mice. (A) Gastrocnemius muscle weight. (B) mRNA expression of Nrf2 signaling. (C) 4‐HNE and Nrf2 target proteins. Data are presented as mean ± SEM. *n* = 6 in each group. *P <* *0.05 **P <* *0.01, significant difference between CON and DEN. ^†^ *P *<* *0.05 ^††^ *P *<* *0.01, significant difference between WT and KO. KO, Nrf2 knockout; 4‐HNE, 4‐hydroxynonenal; CON, sham‐operated control; DEN, denervation; WT, wild‐type.

There was no difference in either body weight or control gastrocnemius muscle weight between WT and KO mice. Two weeks of denervation resulted in a similar decrease in muscle weight in both WT and KO mice (Fig. 1A).

### Nrf2 signaling and oxidative stress

Nrf2 mRNA expression was significantly increased in denervated muscle of WT mice (Fig. 1B). We also observed increases in mRNA expression of Nrf2 target antioxidant genes in WT denervated muscle; however, these increases were attenuated in KO mice (Fig. 1B). To further confirm our findings, we analyzed protein levels of the major Nrf2 targets. Protein levels of Hmox1 and catalase were higher in denervated muscle, while the response was attenuated in KO mice (Fig. 1C). The level of 4‐HNE content, a marker of lipid peroxidation, was robustly elevated in KO denervated muscle (Fig. 1C).

### Mitochondrial content and dynamics

**Figure 2:**
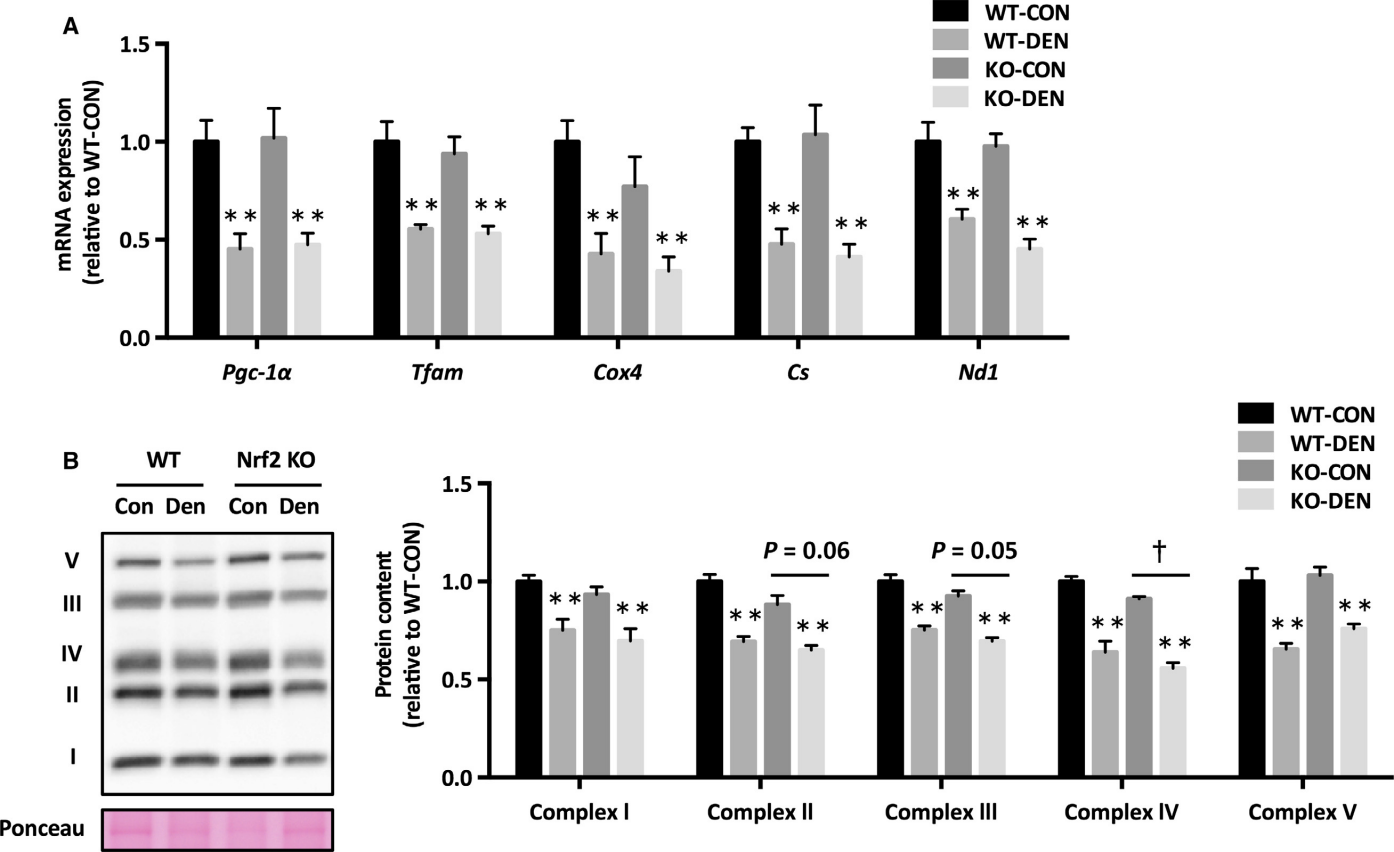
Mitochondrial content following denervation in Nrf2 KO mice. (A) mRNA expression of mitochondrial genes. (B) Mitochondrial oxidative phosphorylation protein content. Data are presented as mean ± SEM. *n* = 6 in each group. **P <* *0.01, significant effect of denervation. ^†^ *P *<* *0.05, significant effect of genotype. KO, Nrf2 knockout; WT, wild‐type; CON, sham‐operated control; DEN, denervation.

**Figure 3:**
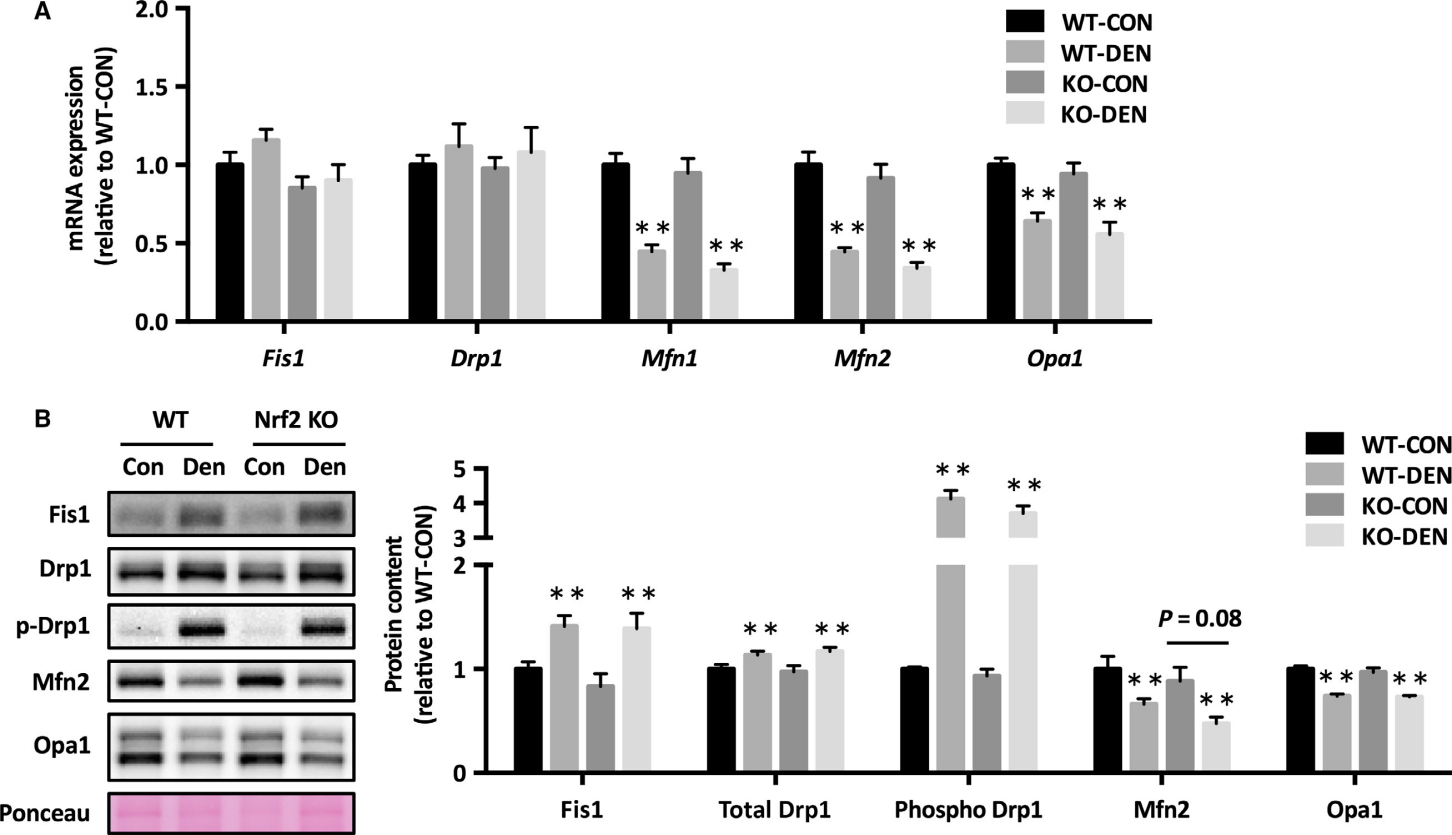
Mitochondrial morphology proteins in response to denervation in Nrf2 KO mice. (A) mRNA expression, (B) protein levels of mitochondrial fusion and fission machinery components. Data are presented as mean ± SEM. *n* = 6 in each group. **P <* *0.01, significant effect of denervation. KO, Nrf2 knockout; WT, wild‐type; CON, sham‐operated control; DEN, denervation.

Denervation resulted in reduced mRNA expression of genes involved in mitochondrial biogenesis (Pgc‐1*α*, Tfam), tricarboxylic acid cycle (Cs), and oxidative phosphorylation (Cox4 and Nd1) (Fig. 2A). The protein level of complex IV was lower in Nrf2 KO mice, and complex II and III approached significance (*P* = 0.05 and 0.06, respectively) (Fig. 2B). We found that mRNA expression of mitochondrial fusion regulatory proteins (Mfn1, Mfn2, and Opa1) decreased after denervation, but there was no change in the mRNA expression of fission regulatory proteins (Fis1 and Drp1) (Fig. 3A). Denervation increased Fis1 protein level and Drp1 phosphorylation, whereas it decreased Mfn2 and Opa1 protein levels (Fig. 3B). There was no effect of Nrf2 deficiency on mitochondrial dynamics proteins, except Mfn2, which approached significance (*P* = 0.08).

### Autophagy

**Figure 4:**
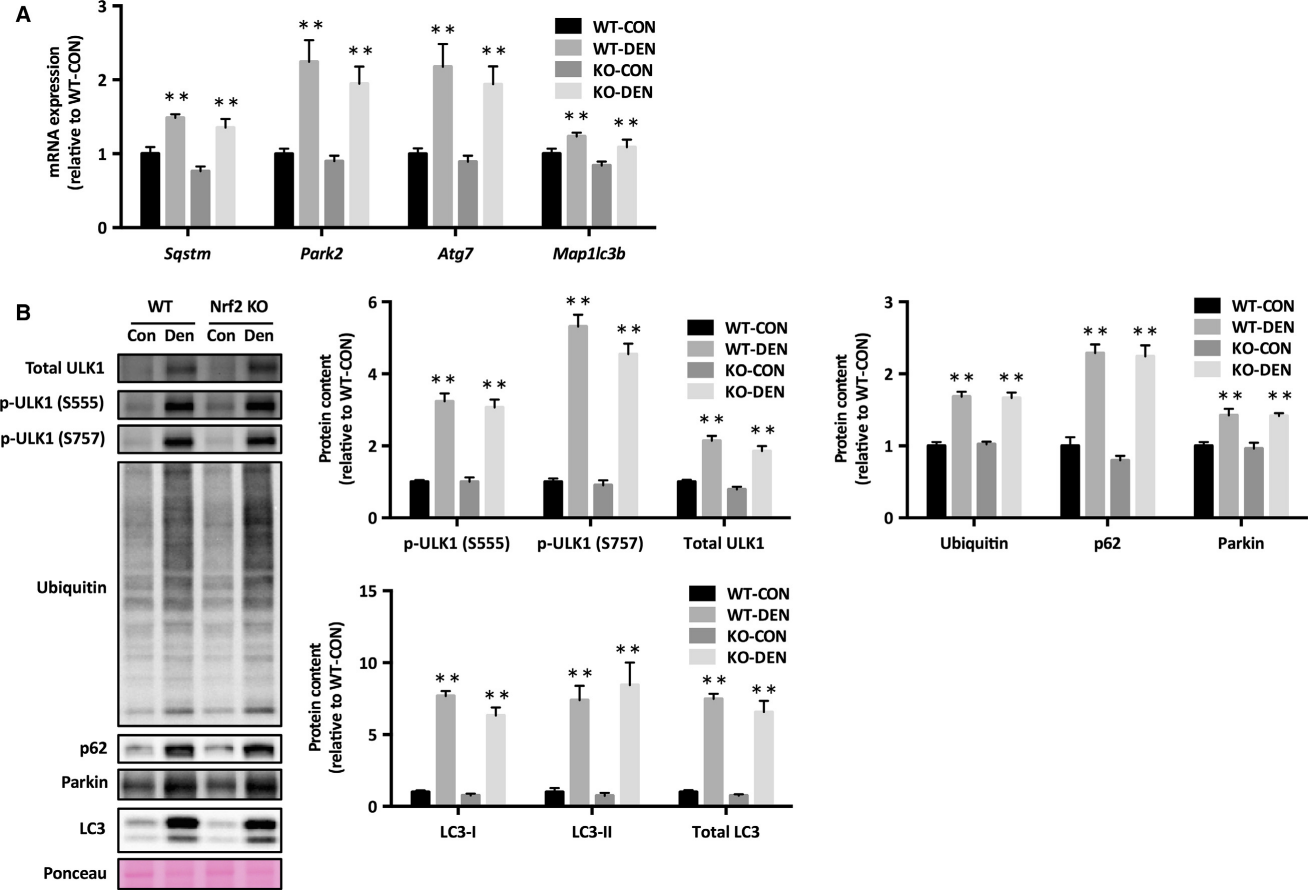
Autophagy in response to denervation in Nrf2 KO mice. (A) mRNA expression, (B) protein levels of autophagic signaling. Data are presented as mean ± SEM. *n* = 6 in each group. **P <* *0.01, significant effect of denervation. KO, Nrf2 knockout; WT, wild‐type; CON, sham‐operated control; DEN, denervation.

Significant increases in mRNA expression of Park2, Atg7, Map1lc3b, and Sqstm were observed in response to denervation (Fig. 4A). Denervation increased the levels of phosphorylated ULK1 at Ser555 and Ser757 (Fig. 4B). Denervation also resulted in the increase in protein levels of LC‐3I, LC‐3II, p62, Parkin as well as of ubiquitin (Fig. 4B). These results indicate that Nrf2 KO does not alter denervation‐induced autophagy.

## Discussion

In this study, we investigated the role of Nrf2 during denervation in skeletal muscle. Our results revealed that Nrf2 signaling was upregulated in response to denervation in WT mice. Nrf2 deficiency downregulated mRNA expression of its target antioxidant genes. The elevated expression of Nrf2 downstream genes in response to denervation in WT mice was attenuated in Nrf2 KO mice. Next, we evaluated oxidative damage in the skeletal muscle. Lack of the compensatory upregulation of Nrf2 signaling robustly enhanced oxidative stress in the skeletal muscle after denervation in Nrf2 KO mice. This observation is consistent with that of previous studies reporting higher oxidative damage in lungs exposed to cigarette smoke (Rangasamy et al. 2004) and in acetaminophen‐treated liver (Enomoto et al. 2001) of Nrf2 KO mice, suggesting that Nrf2‐mediated response counteracts oxidative stress and maintains cellular redox homeostasis.

It has been demonstrated that denervation induces (1) ROS production and oxidative damage (O'Leary and Hood 2008; Abruzzo et al. 2010), (2) mitochondrial fragmentation (Romanello et al. 2010; Iqbal et al. 2013), (3) mitophagy (O'Leary et al. 2013; Tamura et al. 2015), and (4) loss of mitochondrial content and muscle mass (Wicks and Hood 1991; Furuya et al. 2014). In this study, we examined whether Nrf2 deficiency aggravates mitochondrial adaptations in denervated muscle. In contrast to electrical stimulation which increases mitochondrial fusion proteins in skeletal muscle (Iqbal et al. 2013; Kitaoka et al. 2016), we found that denervation increased mitochondrial fission proteins, but decreased fusion proteins. Despite the elevated oxidative damage, however, Nrf2 deficiency did not affect denervation‐induced alterations in mitochondrial fission and fusion proteins in skeletal muscle. We also found that protein levels of mitochondrial OXPHOS tended to be lower in Nrf2 KO mice than in WT mice, but the magnitude of decrease was modest.

Mitochondrial content is determined not only by biogenesis but also by degradation; damaged mitochondria are removed by mitophagy. Intriguingly, the expression of p62, which acts as an autophagic adaptor protein for Parkin‐directed mitophagy (Geisler et al. 2010), is regulated by Nrf2 and at the same time, p62 contributes to activate Nrf2 through a positive feedback loop (Jain et al. 2010; Komatsu et al. 2010). This prompted us to hypothesize that denervation‐induced mitochondrial degradation is attenuated in Nrf2 KO mice. We noted that the expression of the markers of mitophagy was upregulated in response to denervation, in agreement with the findings of previous studies (O'Leary and Hood 2009; O'Leary et al. 2013). However, to our surprise, there was no difference between WT and Nrf2 KO mice. The increase in p62 could be driving the increase in Nrf2 expression in response to denervation in WT mice, but the upregulation of p62 in denervated muscle of Nrf2 KO mice is suggestive of the contribution of other transcription factors.

In this study, we provided evidence that Nrf2 signaling is compensatory upregulated in denervated muscle to counteract oxidative stress, but it has little effect on mitochondrial adaptation. Recent studies have demonstrated that muscle contraction increases Nrf2 expression in the skeletal muscle (Horie et al. 2015; Wang et al. 2016), and Nrf2 is required for exercise training induced mitochondrial biogenesis (Crilly et al. 2016; Merry and Ristow 2016). These results suggest that a pathway independent of Nrf2 may contribute for the maintenance of mitochondrial function during denervation. Interestingly, loss of Nrf2 induced a more striking declines in antioxidant gene expression in aged skeletal muscle than in muscle of young animals (Miller et al. 2012). Further studies are required to investigate the role of Nrf2 in aging‐associated muscle dysfunction. Finally, a limitation of our study is that we did not measure mitochondrial respiratory function. Recent work revealed that the ablation of Nrf2 impaired state 4 respiration rates of intermyofibrillar mitochondria in muscle from Nrf2 KO mice (Crilly et al. 2016). Thus, we cannot eliminate that the modest declines in mitochondrial proteins result in significant respiratory dysfunction.

Here, we showed that denervation‐induced oxidative stress was enhanced in Nrf2 KO mice owing to attenuated upregulation of antioxidant gene expression. However, Nrf2 deficiency did not affect denervation‐induced changes in mitochondrial content, mitochondrial dynamics regulatory proteins, and mitophagy. Our results suggested that Nrf2 deficiency does not exacerbate denervation‐induced mitochondrial dysfunction, and Nrf2 does not play a role beyond regulating target antioxidant gene expression.

## Conflict of Interest

None declared.
